# Using Codesign to Develop a Health Literacy Intervention to Improve the Accessibility and Acceptability of Cardiac Services: The Equal Hearts Study

**DOI:** 10.1111/hex.70328

**Published:** 2025-06-17

**Authors:** Denise Azar, Sofia Wang, Liz Flemming‐Judge, Anna Wong Shee, Rebecca Jessup, Laveena Sharma, Shihoko Fukumori, Jason Talevski, Stephen J. Nicholls, James Harris, Laura Alston, Catherine Martin, Ernesto Oqueli, William van Gaal, Alison Beauchamp

**Affiliations:** ^1^ School of Rural Health Monash University Warragul Australia; ^2^ Victorian Heart Institute Monash University Melbourne Australia; ^3^ Consumer advisor Melbourne Australia; ^4^ Chief Medical Office, Grampians Health Ballarat Ballarat Australia; ^5^ Department of Rural Health, School of Medicine Deakin University Geelong Australia; ^6^ Academic and Research Collaborative in Health La Trobe University Melbourne Australia; ^7^ Allied Health Research Northern Health Melbourne Australia; ^8^ Victorian Heart Hospital Monash Health Melbourne Australia; ^9^ Victorian Virtual Emergency Department, The Northern Hospital Northern Health Melbourne Australia; ^10^ School of Psychology and Public Health La Trobe University Melbourne Australia; ^11^ Department of Medicine–Western Health The University of Melbourne Melbourne Australia; ^12^ Biomedical Manufacturing, Commonwealth Scientific and Industrial Research Organisation (CSIRO) Melbourne Australia; ^13^ Centre for Inflammatory Diseases, School of Clinical Sciences at Monash Health, Faculty of Medicine Nursing and Health Sciences Monash University Melbourne Australia; ^14^ Research Unit, Colac Area Health Colac Australia; ^15^ Department of Epidemiology and Preventive Medicine Monash University Melbourne Australia; ^16^ Cardiology Department Grampians Health Ballarat Ballarat Australia; ^17^ School of Medicine Deakin University Geelong Australia; ^18^ Cardiology Department The Northern Hospital Melbourne Australia; ^19^ Faculty of Medicine The University of Melbourne Melbourne Australia

**Keywords:** accessibility, cardiovascular, codesign, health literacy, intervention development, patient and public involvement

## Abstract

**Introduction:**

The burden of coronary heart disease (CHD) is disproportionately greater among socio‐economically disadvantaged groups. Health services play a crucial role in addressing this social gradient by ensuring equitable access to care. However, there is limited evidence on effective strategies to improve health service accessibility for CHD patients, particularly those that are codesigned with people with lived experience and clinicians. The Equal Hearts study aimed to codesign a health literacy‐based intervention to improve the accessibility of hospital‐based cardiac services for underserved population groups with CHD.

**Methods:**

This study employed a mixed‐methods approach based on codesign principles. The study comprises three phases: identifying and understanding the problem, codeveloping an intervention, and translating the intervention into practice. Phases 1 and 2 are reported in this paper and included focus groups, interviews and an intervention development workshop. Participants for focus groups and interviews were recruited from four health services in [Victoria] and included patients with CHD, health consumers from culturally diverse communities and clinicians. Findings from focus groups and interviews were analysed via thematic analysis using Levesque's conceptual framework to identify health literacy barriers to accessibility of cardiac services. These barriers were prioritised in a codesign workshop with cardiac patients, health consumers and clinicians.

**Results:**

Thirty‐seven cardiac patients, 10 clinicians and 44 culturally diverse health consumers participated in focus groups/interviews. Among these participants, eight cardiac patients/carers and five clinicians attended the workshop. Cardiac patients reported a lack of preparedness for hospital discharge and feeling ‘lost’ and uncertain about how to confidently manage their health at home after a cardiac event. A codesigned intervention—The Patient Discharge Action Plan—aims to improve patients' transition from hospital to home.

**Conclusion:**

Using a codesign approach and health literacy principles, a health service intervention was developed to improve accessibility of cardiac services. The Patient Discharge Action Plan is currently being evaluated in a pilot RCT.

**Patient or Public Contribution:**

Two consumer co‐authors [L.F.J. and J.H.] informed the development of the study protocol. A Stakeholder Advisory Panel, including six people with lived experience of CHD and four clinicians/health service managers from participating sites, guided all steps within this study.

**Trial Registration:**

ACTRN12624000780550p (Australian and New Zealand Clinical Trials Registry). Registered on 25 June 2024.

## 1 Introduction

Coronary heart disease (CHD) is a leading cause of death in Australia and worldwide. From 2018 to 2019, CHD was the primary cause of more than 161,000 hospitalisations in Australia and over $2.4 billion AUD in direct healthcare costs [1]. The burden of CHD is greater among people living with socio‐economic disadvantages, including culturally and linguistically diverse (CALD) communities, rural Australians, those with lower income or education, and people with lower health literacy. Compared with their more advantaged counterparts, these population groups have increased mortality rates [2], higher morbidity rates and greater risks of subsequent cardiac events [3], such as myocardial infarction (MI). Health services, ranging from emergency care, primary care, specialist care, hospital care and preventive care, play an important role in addressing these health inequities by ensuring the care they provide is ‘accessible’ for all population groups. Health service accessibility can be defined as ‘the availability and affordability of services, and their acceptability and appropriateness’ [4, 5]. Health services are seen as accessible if the care they provide ‘fits’ the characteristics and needs of patients [4]. These patient characteristics include the physical and social environments in which they live, as well as their social status and economic resources [4]. Services that are perceived as accessible by patients are associated with stronger relationships with providers and greater usage of the service when needed, leading to improved self‐management and better overall health [6]. However, there is little evidence from consumer perspectives about what makes a service accessible [6] and even less for cardiac patients from vulnerable communities [7, 8].

Supporting patients' health literacy needs may offer a mechanism by which health services can improve their accessibility and enhance patient engagement. Health literacy refers to an individual's ability to find, understand, appraise and use information and services to make decisions about health [9]. Health literacy is a multidimensional concept and includes not only literacy and numeracy skills but also the ability to navigate health services, communicate with health providers and apply health information to everyday life [9]. Health literacy is a determinant of health, and previous research has shown that health literacy is lower among socially disadvantaged groups [10, 11, 12, 13]. For individuals with CHD, low health literacy has been associated with adverse health outcomes, including increased mortality and hospital readmission rates and decreased quality of life [14, 15]. Health literacy interventions that make it easier to navigate health services and engage with providers, as well as those that improve information provision, may improve the accessibility of cardiac services for underserved populations.

The development of interventions that are acceptable, feasible and sustainable requires input from end users, that is, patients and relevant stakeholders [16, 17, 18, 19, 20, 21]. Codesign is a process in which targeted end users and stakeholders form a partnership with researchers and work together on all aspects of intervention development, from needs assessment through content development and pilot testing. In a recent meta‐analysis, codesigned interventions were shown to have positive effects on health behaviours and health service accessibility across multiple health conditions [17]. However, a scoping review of CHD studies confirmed that codesign is still an emerging field, with few studies evaluating links between codesigned CHD interventions and improved patient outcomes, particularly in socio‐economically disadvantaged population groups [22]. There are also few studies involving health literacy‐informed approaches to improve outcomes and health service accessibility for people with CHD [21]. Codesign may offer a robust and practical approach for developing health literacy interventions to improve accessibility to health services that are equitable, acceptable and effective [20, 23, 24]. There is a need to test the potential for codesign as an approach to creating interventions focused on health literacy in CHD management that empower patients to use, navigate and understand the complex information landscape surrounding cardiovascular health.

The Equal Hearts study aims to codesign and evaluate a health literacy intervention to improve the accessibility of hospital‐based cardiac services for underserved population groups with CHD. Our study has four objectives: (1) to understand the perspectives of cardiac patients and clinicians about health literacy‐related factors affecting accessibility, (2) to understand the perspectives of consumers from culturally diverse backgrounds about the accessibility of health services more generally, (3) to identify the priority issues among these groups, and (4) to develop a codesigned, health literacy intervention.

## 2 Materials and Methods

### 2.1 Study Design

**Figure 1:**
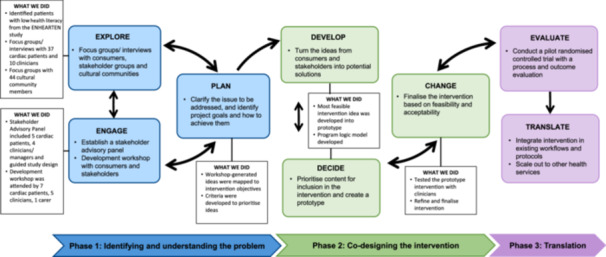
Overview of the study phases and codesign elements in the study. Adapted from the framework of Boyd et al. [25].

The Equal Hearts study is a mixed‐methods study based on codesign principles. In the absence of reporting guidelines for codesign methodology, this study is reported as per the Guidance for Reporting Involvement of Patients and the Public 2—GRIPP 2 short form reporting checklist [25] (See Additional file 1). We utilised an established codesign framework by Boyd et al. [26]. This framework has six steps over two phases: the first three aim to understand the patient experience, and the latter three aim to improve that experience through action. Our application of the framework is provided in Figure 1. We have added a third phase (‘Translation’), comprising ‘Evaluate’ and ‘Translate’. Although often depicted as a linear progression of stages, codesign is, in practice, a dynamic and iterative process. As Boyd et al. [26] emphasise, the crux of codesign lies not in adhering to a rigid sequence but in ensuring active consumer involvement throughout each component of the process. This fluidity and continuous engagement are essential characteristics that define the true essence of codesign. While the Equal Hearts study encompasses three phases, as described above, this paper describes the first two phases.

For the purposes of this study, hospital‐based cardiac services span first contact through to the first 12 months after a CHD event, including emergency departments (EDs) and inpatient and outpatient services.

### 2.2 Setting and Participants

This multicentre study was based in Victoria, Australia, across two metropolitan and two rural health services (defined using MMM categories) [27]. The participants in the following two groups were recruited for this study.

1. *Patients and clinicians from cardiac services within one of the above health services who participated in one or more of the following activities: focus groups/interviews, the Stakeholder Advisory Panel, and a development workshop*. Patients were recruited from an existing prospective cohort study (ENHEARTEN), which aims to explore associations between baseline health literacy and cardiac outcomes in people with their first MI [28]. Participants in ENHEARTEN were recruited from three of the above health services. ENHEARTEN participants with low health literacy (low scores on the Health Literacy Questionnaire [29]) who agreed to be contacted for further research were identified and approached by the research team via email, post or telephone and invited to participate in a focus group. In addition, eligible patients from cardiac outpatient services were identified through patient lists and contacted by members of the research team, who were staff members within each health service. Inclusion criteria were aged over 18 years with a diagnosis of CHD, medically well enough to participate (or their carer) and able to understand English. Eligible clinicians/managers working within cardiac services at each of the four participating sites were invited by email to a focus group or interview.
2. *Health consumers from CALD communities (African, Sikh and Arabic‐speaking) for focus groups*. These communities were selected because they have high rates of cardiometabolic disease [30] and are among the priority population groups for health services. The participants were identified via community organisations, associations and places of worship. We contacted these organisations via email or telephone and invited them to disseminate expressions of interest to their eligible community members. Given that there may be cultural sensitivities toward taking part in research studies, the recruitment process was undertaken in collaboration with staff from the above community organisations [31]. Inclusion criteria for these cultural community members were being over 18 years of age; having experience using health services within Australia; providing informed consent; and understanding English, Arabic, Dinka or Punjabi. Recruitment materials were translated as needed, and interpreter services were engaged to support the consent process, if needed.

### 2.3 Phase 1: Identifying and Understanding the Problem

#### 2.3.1 Explore

##### 2.3.1.1 Focus Groups/Interviews

Focus groups and interviews with cardiac patients and clinicians were conducted across the four health services, either face‐to‐face or via Zoom. Following each focus group and interview, we asked participants to indicate their interest in taking part in a Stakeholder Advisory Panel to guide each phase of this study. For CALD consumers, three focus groups were undertaken with the three cultural community groups. Each focus group lasted up to 50 min and was facilitated by one or two co‐authors (A.B., D.A. and R.J.), all of whom are experienced qualitative researchers with previous experience of facilitating focus groups among people living with chronic disease. A.B. is also a former cardiac nurse. Where possible, focus groups with cultural communities were co‐facilitated by a consumer researcher (L.F.J.) who had considerable experience working with culturally diverse populations. None of the facilitators had any previous relationship with the participants. In‐person, accredited interpreters were used for focus groups with cultural communities where required. Following Braun and Clarke's reflexive thematic analysis approach [32], we did not use data saturation to determine the sample size but instead focused on collecting data that was sufficient to generate rich, meaningful interpretations addressing our research objectives. Focus group topics were developed, drawing on elements of Levesque's Conceptual Framework of Access to Health [4] and the health literacy literature. The topics for discussion included participants' experiences accessing hospital‐based services in Victoria and whether the services met their health literacy needs. Specifically, participants were asked if these services were easy to use, if the information they provided was useful and easy to understand, and how difficult or easy it was for patients to communicate their needs. Cardiac patients were asked to reflect upon this in relation to cardiac services, whereas CALD consumers were asked to reflect more generally about health service use. Focus groups/interviews were recorded and transcribed. Where required, focus group recordings from cultural communities were transcribed in the language and then translated into English by accredited interpreters. This translation was undertaken independently and did not involve interpreters from the focus groups. Focus group and interview data from all participants were analysed via an inductive, reflexive thematic approach [32] to develop key themes that describe common barriers and enablers of service accessibility. Detailed methods and findings from the cultural community focus groups and the focus groups/interviews with cardiac patients and clinicians will be published separately. All patients/consumers who participated in a focus group or interview were offered a $20 gift voucher.

#### 2.3.2 Engage

##### 2.3.2.1 Stakeholder Advisory Panel

A Stakeholder Advisory Panel was established to guide aspects of the study, including five patients with lived experiences of cardiac disease and four clinicians/managers who worked within cardiac services. For patients, we aimed to incorporate diversity wherever possible on the basis of age, sex, rurality and cultural background. The Stakeholder Advisory Panel met approximately 5–6 times over the project period via Zoom, with email and phone contact as needed. In line with codesign principles, the Stakeholder Advisory Panel meetings were cochaired by a consumer investigator (L.F.J.), who also contributed to the overall design of this study. Within most meetings, there was an opportunity for ‘break‐out’ rooms where patients could meet separately with L.F.J. to discuss any issues they were not comfortable raising in the wider group. Patient panel members were remunerated for their involvement. The initial meeting with the Stakeholder Advisory Panel involved discussing the purpose of the study and establishing the terms of reference. A subsequent meeting was held to discuss the findings of the focus groups/interviews and to commence planning for the workshop. Members of the Stakeholder Advisory Panel were also invited to attend the development workshop.

##### 2.3.2.2 Development Workshop

We held a face‐to‐face development workshop on 9 February 2024, in Melbourne, Australia. The purpose of the workshop was to consider findings from Phase 1, prioritise the main issues and identify potential solutions and ideas to address those priorities. Participants included cardiac patients, carers and clinicians. Most of these individuals had previously engaged in the focus groups during Phase 1 and/or served as members of the Stakeholder Advisory Panel. As the workshop aimed to identify ways to improve the accessibility of cardiac services, we did not include participants from the cultural focus groups, as most lacked direct experience with cardiac services. However, their insights into barriers to accessibility were included as part of the workshop activities, and workshop participants included rural participants and those with low health literacy. Findings from the cultural focus groups were reported to each cultural group, and discussions were held about the implications of these findings for each community and the potential for future codesign work to improve the cultural acceptability and accessibility of health services (separate from this study).

During the development workshop, participants were assigned to three small groups, each comprising a mix of patients and clinicians from different health services, age groups and rural or metropolitan origins. The workshop activities are described in Table 1. Workshop participants provided written consent, and patients were offered a $20 gift voucher. Patient Stakeholder Advisory Panel members were remunerated for their time.

**Table 1:** Workshop activities undertaken.

Workshop activity	Description of activity
1. Understanding the barriers to accessibility	A ‘vignette’ (Figure 2) that described a fictional character with their first MI, who represented key socio‐demographic characteristics and had experienced several of the barriers identified from focus group data, was presented. In small groups, participants discussed the vignette and focus group findings (Figure 3) and were asked to identify reasons underlying the barriers that the character experienced. This step allowed participants to become familiar with the data in greater depth and consider the broader context around each barrier.
2. Prioritising the issues	Participants were asked individually, ‘What do you see as the most important issue to address?’ and given coloured star stickers to place next to each issue listed on the patient journey map. Gold stars represented the most important issues, silver stars for the second and bronze stars for the third most important issues to be addressed. Stars were assigned points to determine the most important issues (gold = 3, silver = 2 and bronze = 1). The points were summed for each statement, and the two highest scoring issues confirmed by participants as the most important issues (Table 2).
3. Generating solutions and ideas	Participants reconvened in their groups to generate potential ideas and solutions to address the identified priorities from the previous activity. Participants were each provided with an activity sheet to brainstorm potential solutions, which were then shared within their group and, later, in the broader group (Table 3).
4. Choosing the best idea	In the final activity, the broader group was asked what criteria could be used to select the ‘best’ idea (e.g., feasibility and cost). They were also asked whether any ideas could fit together to form one overarching intervention. Scribes noted the points raised in this discussion, which were then considered during the subsequent process of intervention development (see Additional file 2).

**Figure 2:**
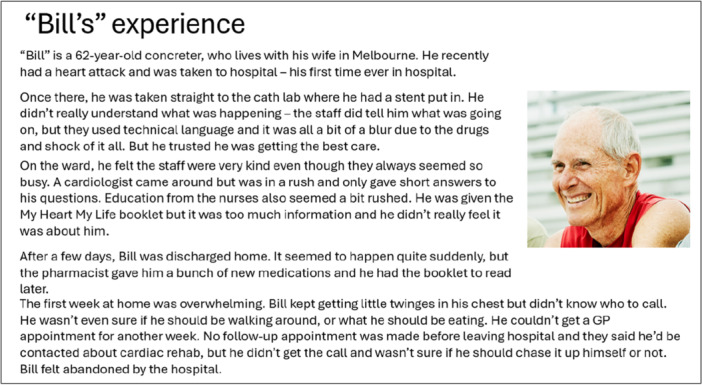
Vignette about ‘Bill's’ experience with a cardiac service shown at the workshop.

#### 2.3.3 Plan

Following the development workshop, the planning stage involved engaging with the Stakeholder Advisory Panel to clarify the issue/s and identify project goals and how to achieve them. A comprehensive summary of the workshop proceedings and discussions was disseminated to all attendees, seeking their validation of the research team's interpretations. Selection of the most appropriate intervention was an iterative process. First, the researchers developed intervention objectives, that is, the desired changes that an intervention should be expected to achieve, on the basis of the findings from the *Explore* stage. The workshop‐generated intervention ideas were then mapped against these objectives to help select the more promising ideas. The selection criteria developed by workshop participants and refined by the researchers were also used to prioritise those ideas most likely to be acceptable and successful (see Table 4). Next, a rapid literature review was conducted to determine the effectiveness of the key intervention ideas that were starting to emerge. Discussions with clinical leads at the lead site also informed the feasibility of key intervention ideas. From these steps, five key ideas for the intervention were identified. These key ideas were discussed with the Stakeholder Advisory Panel to determine the ‘best’ intervention to take forward for development, considering what was feasible within the project's scope.

### 2.4 Phase 2: Codesigning the Intervention

#### 2.4.1 Develop and Decide

Based on discussions with the Stakeholder Advisory Panel and the cardiac services team, the researchers developed the intervention solution deemed most feasible and implementable into a prototype. To further refine this intervention, the Stakeholder Advisory Panel convened to construct a programme logic model. This model delineated the intervention's objectives and design, outlined the resources necessary for conducting the pilot RCT, specified the anticipated outputs, and proposed methods for evaluating short‐term and medium‐term outcomes. This structured approach ensured a well‐defined and thoroughly considered intervention strategy.

#### 2.4.2 Change

The cardiac rehabilitation team tested the intervention prototype to gather final feedback from the clinicians who will deliver the intervention in practice. This feedback was used to finalise the intervention for its feasibility and acceptability. The culmination of this phase was a codesigned intervention ready for testing in Phase 3.

## 3 Results

### 3.1 Results From Phase 1—Identifying and Understanding the Problem

**Figure 3:**
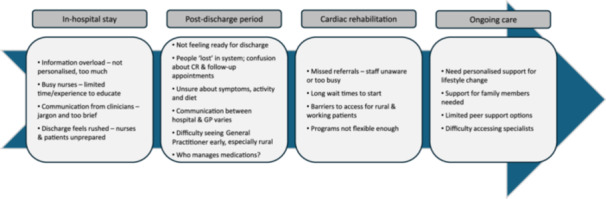
Patient journey map presented at the development workshop summarising common experiences and issues related to accessibility to cardiac services from the focus groups and interviews organised into the different stages of a patient's care after a cardiac event.

Focus groups and interviews were conducted with 37 cardiac patients, 10 clinicians and 44 CALD consumers. A summary of the issues identified is provided in Figure 3.

Development workshop participants included seven cardiac patients, five clinicians and one carer. In the workshop, participants were asked to prioritise the barriers to accessibility that an intervention should aim to address. Table 2 provides the top five rated barriers to accessibility to cardiac services as determined by workshop participants. Each statement was categorised according to the stage of care outlined in the patient journey map. Participants identified a total of 27 ideas to address the barriers to accessibility (Table 3).

**Table 2:** Top five rated issues related to accessibility to cardiac services from the development workshop.

Problem/issue	Category of care	Score
1. Discharge feels rushed—nurses and patients unprepared	In‐hospital stay	17
2. People ‘lost’ in system; confusion about cardiac rehabilitation and follow‐up appointments	Post‐discharge period (1–2 weeks)	16
3. Difficulty accessing specialists	Ongoing care	16
4. Limited personalised support for lifestyle change	Ongoing care	12
5. Information overload—not personalised, too much in a short time	In‐hospital stay	10

**Table 3:** Workshop‐generated ideas and solutions to improve barriers to accessing cardiac services.

Problem/issue	Potential solutions and ideas from the workshop
Lack of communication between clinicians and patients	Dedicated time in‐hospital with a nurse educator to speak about the patient's condition or progress Patient advocate in‐hospital to liaise between patients and clinicians Multiple avenues to receive information in‐hospital (i.e., videos at bedside, existing staff, written information and AI education) Pharmacist to talk to patients in‐hospital
Patients don't know what to ask before going home	Dedicated time to sit with patients regarding education, discharge and post‐discharge information, and time to answer questions Discharge information or Q&A sessions
Patients are not prepared to go home	Clear individualised action plans or checklists on discharge that outline accurate expectations and the next steps to take Planned or staged discharge process; flow chart or checklist Empower patients by providing tools to plan and enact their own discharge journey (access to information, research and advice)
Patients are not educated about their medication when they are discharged home	Patients provided with medication list with the reason for taking the medication and common side effects before discharge
Patients don't know what to do or who to contact after discharge	Tailored information for patients that better accommodates their unique condition Multiple ways to receive information at home (i.e., better use of patient portal, ‘patient advice’ websites, videos and phone line) Patients were provided with a contact number for any questions post‐discharge Centralised information hub Include the availability of a GP management plan and the ability to access allied health services in the patient information Patients are given more opportunities to interact with peers at cardiac rehabilitation and support one another Arrange transportation for rural patients after discharge
Patients become ‘lost’ in the system—no follow‐up	Discharge follow‐up phone service Volunteer phone/internet support chat groups or online peer support groups (with specific groups for those of CALD backgrounds) Establish processes for early access to cardiac rehabilitation so patients feel supported and have access to education Arrange regular GP or clinician follow‐up appointments for patients
Clinicians in hospital care teams have varying levels of experience in treating cardiac patients	Training for healthcare professionals about patient expectations of care delivery and about providing consistent advice
Clinicians struggle with balancing clinical productivity and responsibility	Improve the patient‐to‐nurse/doctor ratio to give more time for clinicians to talk with patients
Confusion around the responsibilities of the GP and the hospital (i.e., unclear guidelines around the referral process to cardiac rehabilitation)	Cardiac rehabilitation referral and GP appointments made by hospital before discharge Liaison contact number for GPs to call cardiologists if they have questions Establish standardised processes across health services
Medical discharge summaries are incomplete or confusing	Discharge summaries to be uploaded to My Health Record

Abbreviations: AI = artificial intelligence, CALD = culturally and linguistically diverse, GP = general practitioner, Q&A = question and answer.

### 3.2 Results From Phase 2—Codesigning the Intervention

The programme logic model used to describe the development of the intervention, including the resources needed, the activities to be undertaken and the expected outcomes for the intervention, is shown in Additional file 2.

Selection of the intervention was based on the criteria devised by participants in the workshop (Table 4). The proposed intervention, as determined by the Stakeholder Advisory Panel, was a paper‐based Patient Discharge Action Plan (Additional file 3) that aims to help patients feel prepared for discharge and feel better able to manage their health at home. The two‐page action plan ensures that patients know the next steps following discharge (such as following up with a general practitioner and specialist and being referred to cardiac rehabilitation), what to do in the event of chest pain and what to do regarding their medication. The action plan is also personalised for each patient with a section that addresses the patient's individual concerns (e.g., about driving, exercise, work, etc.) and a section addressing any questions they may have. Links and QR codes to online resources with information about recovery and cardiac rehabilitation (from the National Heart Foundation and lead site) are also provided.

**Table 4:** Co‐created criteria for selecting an intervention idea.

Criteria
Easy to understand
Able to be tailored/applicable to many people (e.g., equitable) and can be used by people from metropolitan and rural/regional areas
Able to be used by other health services
Cost‐effective
Able to achieve both ‘early’ wins and long‐term wins

### 3.3 Results From Phase 3—Translation

To facilitate changes in practice patterns, we will work closely with health service managers and clinical leaders to integrate the intervention into existing workflows and protocols. Regular feedback sessions and continuous monitoring allow for iterative improvements and ensure the intervention's sustained adoption. Furthermore, if positive outcomes are observed, the possibility of scaling up the intervention to include the three other health services in the Equal Hearts study may be explored. This expansion would involve adapting the intervention through codesign to suit the specific context and needs of each health service while maintaining the core principles of our health literacy‐based approach. The final details of the intervention according to the TIDieR checklist [33] are reported in Additional file 4.

## 4 Discussion

Given the role of low health literacy in CHD patient outcomes, this study codesigned a health literacy intervention aimed at improving the accessibility of cardiac services for patients. By leveraging codesign methods, we created a health literacy intervention to empower patients to navigate, understand and effectively use the complex information landscape of cardiovascular health. This approach fills a significant gap in the CHD care literature and ensures that the intervention is tailored to patients' needs, potentially leading to improved health outcomes and more equitable access to cardiac services.

To date, two studies have used a codesign process to develop health literacy‐based interventions to improve outcomes for CHD patients [16, 34]. Aaby et al. [16] used the Ophelia (OPtimising HEalth LIteracy and Access) codesign methodology to improve organisational health literacy responsiveness in cardiac rehabilitation, producing a set of feasible interventions targeting vulnerable populations. Toledo‐Chávarri et al. [34] used a codesign approach to produce a patient journey map that helped patients with CHD feel empowered during the diagnosis, post‐diagnosis and long‐term care stages. However, neither of these studies reported detailed methods for establishing meaningful relationships with consumers and stakeholders. This relates to the *Engage* element of Boyd et al.'s [26] codesign framework, which emphasises the importance of genuine engagement with consumers and stakeholders throughout the process. Our study has applied the principles of codesign methodology, including the establishment of a Stakeholder Advisory Panel, to gain richer insights from those with lived experience of utilising and working in cardiac services.

The Patient Discharge Action Plan addresses the key issues identified through the first two phases of the study: patients feeling lost after discharge from the hospital and receiving too much generic information while in the hospital. Most hospitals have established discharge planning guidelines to ensure continuity of care. In accordance with the current study's lead site guidelines, bedside nurses must provide comprehensive discharge instructions and allied health staff should arrange follow‐up services. While verbal instructions are important, patients require written discharge plans to reinforce and remember their ongoing care instructions [35]. Ideally, discharge plans should include personalised instructions for patients and subsequent healthcare providers. In Australia, patients with CVD conditions are often provided with educational resources to support their recovery and ongoing health management. However, despite the availability of such resources, patients in this study reported feeling unprepared to leave the hospital and being unsure of post‐discharge steps, a finding reported in several other studies [36, 37]. Common reasons included not receiving information or finding the information too overwhelming to process. This information gap often led to patients not receiving necessary follow‐up care, potentially hindering their recovery. The Patient Discharge Action Plan alleviates these issues by ensuring that patients are not lost to follow‐up. Implementing the action plan would mean that follow‐up appointments with the patient's specialist and referrals to cardiac rehabilitation are arranged before discharge. Additionally, providing patients with a phone number allows them to reach the hospital with any questions after discharge. Careful consideration was given to its design from a health literacy perspective, including plain language, colour selection and text font size. The Stakeholder Advisory Panel reviewed and provided feedback on these aspects, ensuring that the plan was accessible and easy for all patients to understand. The single‐sheet design was deliberately created to prevent information overload, allowing patients to easily reference their post‐discharge instructions at a glance. Similar strategies in the United States, such as IDEAL discharge planning and the re‐engineered discharge (RED) toolkit, have been shown to be effective in reducing readmissions and post‐hospital ED visits [38, 39, 40, 41, 42, 43].

### 4.1 Strengths and Limitations of This Study

The use of a codesign methodology to develop a health literacy intervention is a major strength of this study. The importance of engagement with people with lived experience is widely acknowledged as essential in the delivery of quality healthcare, and it is also highly recommended in health services research [44]. This study placed high value on consumer and end‐user inputs, aiming to validate barriers and enablers to cardiac service accessibility before intervention development. By understanding both patient and clinician experiences, the intervention was designed to directly address their needs. The participants generated a collection of ideas for improving issues related to the accessibility of cardiac services. Since the intervention stemmed directly from their ideas, participants were more likely to participate in the codesign process. Thus, we hypothesise that their consistent collaboration in the design of the intervention increases the likelihood that the general population will accept the intervention. Furthermore, by ensuring that both patients and clinician participants contributed to all steps of the codesign process, the resulting health literacy intervention could consider both the individual and organisational components of health literacy. Other studies that include both cardiac patients and health professionals in the codesign process reported positive responses to their developed intervention [16, 34], suggesting that our intervention may be well‐received by cardiac patients and clinicians.

Limitations should also be considered. As previously mentioned, there was minimal CALD representation in the codesign process of intervention development. Ideally, there should be maximum diversity within consumer engagement groups to capture a wide range of experiences. The perspectives of different CALD populations in the codesign process may have provided more or different specificities that were not collected before intervention development. Therefore, the lack of CALD participation should be considered when the study findings are interpreted. Although great care was taken to ensure the collaborative nature of the codesign process, as with all qualitative research, there may be some discomfort that was not made known to the research team. This may have led to some participants feeling uncomfortable speaking out or providing their perspectives. It is therefore possible that some missed considerations were made in the intervention design. Furthermore, although the Action Plan was provided in hard copy, it included website links and QR codes directing users to online resources about recovery and cardiac rehabilitation. This approach assumes a level of digital literacy and access to technology, which may limit the usefulness of these resources for some patients.

Several key challenges have been identified in the codesign process with people with lived experience in healthcare. These include insufficient engagement of priority populations, predetermined codesign approaches established before project initiation, and the exclusion of people with lived experience from the process, which is often dominated by healthcare professionals or researchers [45]. The following mitigation strategies were used in this study: involving diverse end users, including a Stakeholder Advisory Panel, arranging meetings in spaces other than health services to ensure comfort and safety, using a value‐based approach (i.e., researchers and health services tailor the intervention on outcomes that matter to patients/consumers and health professionals), appointing a person with lived experience as an advisory panel cochair, and not predetermining the intervention to be developed [45].

## 5 Conclusions

Using a codesign approach and health literacy principles, a health service intervention was developed to improve the accessibility of cardiac services. The Equal Hearts Patient Discharge Action Plan is currently being evaluated in a pilot RCT. The Action Plan aims to provide a concise, easily accessible tool for patients to reference their post‐discharge instructions, improve health literacy and reduce complications during the recovery process. By simplifying complex medical information and implementing clear follow‐up protocols, this intervention presents a significant opportunity to enhance the effectiveness and safety of discharge planning.

## Author Contributions

**Denise Azar:** formal analysis (lead), methodology (supporting), project administration (lead), writing – original draft (lead), visualisation (lead), writing – review and editing (equal). **Sofia Wang:** formal analysis (supporting), visualisation (supporting), writing – original draft (supporting), writing – review and editing (equal). **Liz Flemming‐Judge:** conceptualisation (supporting), funding acquisition (supporting), methodology (supporting), writing – review and editing (equal). **Anna Wong Shee:** conceptualisation (supporting), funding acquisition (supporting), methodology (supporting), writing – review and editing (equal). **Rebecca Jessup:** conceptualisation (supporting), funding acquisition (supporting), methodology (supporting), writing – review and editing (equal). **Laveena Sharma:** conceptualisation (supporting), funding acquisition (supporting), methodology (supporting), writing – review and editing (equal). **Shihoko Fukumori:** writing – review and editing (equal). **Jason Talevski:** conceptualisation (supporting), funding acquisition (supporting), methodology (supporting), writing – review and editing (equal). **Stephen J. Nicholls:** conceptualisation (supporting), funding acquisition (supporting), methodology (supporting), writing – review and editing (equal). **James Harris:** conceptualisation (supporting), funding acquisition (supporting), methodology (supporting), writing – review and editing (equal). **Laura Alston:** conceptualisation (supporting), funding acquisition (supporting), methodology (supporting), writing – review and editing (equal). **Catherine Martin:** conceptualisation (supporting), funding acquisition (supporting), methodology (supporting), writing – review and editing (equal). **Ernesto Oqueli:** conceptualisation (supporting), funding acquisition (supporting), methodology (supporting), writing – review and editing (equal). **William van Gaal:** conceptualisation (supporting), funding acquisition (supporting), methodology (supporting), writing – review and editing (equal). **Alison Beauchamp:** conceptualisation (lead), formal analysis (supporting), funding acquisition (lead), methodology (lead), project administration (supporting), writing – original draft preparation (supporting), writing – review and editing (equal).

## Ethics Statement

Ethics amendments were sought to use ENHEARTEN data for this study from the four health services. Ethics approval for this study was received from the relevant human research ethics committee (HREC) at each of the participating health services (lead site Monash Health HREC; approval number: RES‐23‐0000‐007A). Separate ethics approval from the lead site's HREC (RES‐24‐0000‐359B) was obtained for the pilot RCT.

## Consent

All participants provided written informed consent before participating in the study.

## Conflicts of Interest

The authors declare no conflicts of interest.

## Supporting information

Additional File 1. Guidance for Reporting Involvement of Patients and the Public 2 ‐ GRIPP 2 short form reporting checklist.

Additional file 2. The program logic model used to develop the intervention.

Additional File 3. The codesigned, paper‐based Patient Discharge Action Plan.

Additional file 4. TIDieR checklist for the Patient Discharge Action Plan Intervention.

## Data Availability

The datasets used and/or analysed during the current study are available from the corresponding author upon reasonable request.
