# Supplementary material for: Using Codesign to Develop a Health Literacy Intervention to Improve the Accessibility and Acceptability of Cardiac Services: The Equal Hearts Study
Source: Health Expect. 2025 Jun 17;28(3):e70328. doi: 10.1111/hex.70328 (PMC12174474; doi:10.1111/hex.70328)
Supplement: Supplementary file 1 — Additional File 1. Guidance for Reporting Involvement of Patients and the Public 2 ‐ GRIPP 2 short form reporting checklist. [file HEX-28-e70328-s004.docx]

Additional File 1. Guidance for Reporting Involvement of Patients and the Public 2 - GRIPP 2 short form reporting checklist

| **Section and topic** | **Item** | **Reported on page No** |
| --- | --- | --- |
| 1: Aim | Report the aim of PPI in the study | 3-5 |
| 2: Methods | Provide a clear description of the methods used for PPI in the study | 4-10 |
| 3: Study results | Outcomes – Report the results of PPI in the study, including both positive and negative outcomes | 10-12 |
| 4: Discussion and conclusions | Outcomes – Comment on the extent to which PPI influenced the study overall. Describe positive and negative effects | 12-15 |
| 5: Reflections / critical perspective | Comment critically on the study, reflecting on the things that went well and those that did not, so others can learn from this experience | 14-15 |
