# Supplementary material for: Using Codesign to Develop a Health Literacy Intervention to Improve the Accessibility and Acceptability of Cardiac Services: The Equal Hearts Study
Source: Health Expect. 2025 Jun 17;28(3):e70328. doi: 10.1111/hex.70328 (PMC12174474; doi:10.1111/hex.70328)
Supplement: Supplementary file 2 — Additional file 2. The program logic model used to develop the intervention. [file HEX-28-e70328-s003.docx]

Additional file 2. The program logic model used to develop the intervention

| Goal: Patients are prepared at discharge and feel confident in managing their care in the long-term | | | | | |
| --- | --- | --- | --- | --- | --- |
| Problem statement | **Inputs** | **Outputs: Activities** | **Outcomes** | | **Long-term impacts** |
|  |  |  | **Short-term** | **Medium-term** |  |
| Discharge is too rushed  Patients are not prepared to go home  Patients have no support once they are home  Patients get ‘lost’ in the system (referrals, follow-up appointments) | Patients  Family members, friends, carers  Hospital staff (i.e., nurses, doctors, specialists, GPs)  CR staff  Grant funding  Victorian Heart Hospital | Patient Discharge Action Plan  Follow-up appointments made by hospital prior to leaving  Bank of resources addressing: medication, lifestyle factors (physical activity, diet), CR  Staff training (communication with patient, discharge process) | Patients know what to do after discharge  Patients know who to contact after discharge  Patients are not overwhelmed with information during their stay in-hospital – they understand the information provided  Patients are informed about their care in-hospital  Patients are equipped with or have access to enough resources and information before they leave hospital  Resources provided are easy to read and access  Patients are satisfied with their care  Patients have reduced anxiety during their hospital stay  Patients are educated about their medications  Patients know what to ask while they are in-hospital; they can advocate for themselves  Patients are referred to CR before they are discharged  Patient’s individual needs are addressed  Nurses are more confident in discharging patients  Patients know how to modify lifestyle factors to reduce their risk of CHD | Patients have improved health literacy  Patients have increased confidence to interact with health professionals  There are reduced presentations to the emergency department  Increased attendance at CR  Patients know how to manage their medications  GPs are more confident in their responsibilities to the patient  Patients know what to do if they have chest pain or angina  Patients know who to contact if they have questions  Patients can navigate the health system  Patients are proactive in their care  Carers (family members, friends, external) know what to do and how to help the patient manage their condition | Patients are confident in managing their cardiac condition in the long-term  Patients feel supported by the health system  There are consistent processes and guidelines around discharging patients |
| Assumptions: 1) Patients are provided with easy-to-manage, nonoverwhelming resources. 2) Patients develop and are equipped with the skills to advocate for themselves. 3) Patients have some degree of digital literacy and access to digital technologies to get the most out of all aspects of the resource. 4) Discharge nurses/staff are confident in discharging patients. | | | **External factors:** 1) Availability of staff (i.e., busy staff, high turn-over of staff may affect the efficiency of implementing the intervention). 2) Availability of general practitioners and specialists for follow-up appointments. 3) Wait times for CR. 4) Patient health insurance status – may influence the patient’s decision to attend specialist appointments/CR. 5) Perceptions of CR (may be due to cultural differences, personal values, motivation). | | |

CR=cardiac rehabilitation; CHD=coronary heart disease; GP=general practitioner
