# Supplementary material for: Using Codesign to Develop a Health Literacy Intervention to Improve the Accessibility and Acceptability of Cardiac Services: The Equal Hearts Study
Source: Health Expect. 2025 Jun 17;28(3):e70328. doi: 10.1111/hex.70328 (PMC12174474; doi:10.1111/hex.70328)
Supplement: Supplementary file 3 — Additional File 3. The codesigned, paper‐based Patient Discharge Action Plan. [file HEX-28-e70328-s001.pdf]

GOING HOME AFTER A HEART ATTACK
Your discharge action plan

Monash Health

Equal Hearts

If you have chest pain/angina

1. Stop & Sit
2. Use angina spray under your tongue
3. Tell someone about your symptoms
4. No relief after 5 minutes? Use spray again
5. No relief after another 5 minutes? Call 000 for an ambulance

Follow up appointments

See your GP within one week after discharge

Your specialist appointment with:

is on / /

or will be sent to you within 2 weeks.

If no appointment made within 2 weeks, call

Cardiac rehabilitation

You have been referred to

for cardiac rehab.

They will contact you.

If you don't hear from them within 4 weeks, call

Your medications

The hospital Pharmacist has given you a list of your medications. Use this list to:

- Know when to take your medications.
- Take to your GP so they know which medications you are on.

Do not stop taking any medication unless your Doctor or Pharmacist tells you to.

DRAFT

Advice on things that matter to you (Such as driving, work, exercise)

For more information, scan the QR codes below with your mobile phone or visit the links provided.

Your Monash Health Patient Portal (personal information about your heart).

https://victorianhearthospital.org/patients-and-visitors/patient-portal/

General Information about heart attack and cardiac rehab.

http://www.monash.edu/rural-equal-hearts

My notes / questions

Patient information

Name:

UR number:

Hospital admission date: / /

Reason for admission:

In hospital procedure(s) and date(s):

Date of discharge: / /

DRAFT
