# Supplementary material for: Using Codesign to Develop a Health Literacy Intervention to Improve the Accessibility and Acceptability of Cardiac Services: The Equal Hearts Study
Source: Health Expect. 2025 Jun 17;28(3):e70328. doi: 10.1111/hex.70328 (PMC12174474; doi:10.1111/hex.70328)
Supplement: Supplementary file 4 — Additional file 4. TIDieR checklist for the Patient Discharge Action Plan Intervention. [file HEX-28-e70328-s002.docx]

Additional file 4. TIDieR checklist for the Patient Discharge Action Plan Intervention

| **TIDieR Item** | **Description** |
| --- | --- |
| 1. **BRIEF NAME**   Provide the name or a phrase that describes the intervention | Equal Hearts Patient Discharge Action Plan |
| 1. **WHY**   Describe the rationale, theory, or goal of the elements essential to the intervention | Researchers collaborated with patients and clinicians in the Equal Hearts study to codesign health literacy intervention ideas that aim to improve accessibility to cardiac services. The Patient Discharge Action Plan was developed based on an intervention idea suggested by participants in our study. The Stakeholder Advisory Panel and health service staff were consistently involved in the design of the Action Plan. The 1-page Action Plan is designed to ensure that CHD patients know the next steps following discharge based on the information they are given as part of usual care. The Action Plan is also personalized for each patient with a section that addresses the patient’s individual concerns and a section addressing any questions they may have. Expected outcomes are that the intervention group will have a greater improvement in health literacy, lower anxiety and depression, emergency department presentations and higher attendance at cardiac rehabilitation compared to the control group at follow-up time-points. |
| 1. **WHAT: Materials**   Describe any physical or informational materials used in the intervention, including those provided to participants or used in intervention delivery or in training of intervention providers. Provide information on where the materials can be accessed | Clinician training manual   - Study overview and rationale - Recruitment and consent - Data collection procedures   Patient Discharge Action Plan   - The intervention group will receive a double-sided A4 resource that includes personalized, written information about patient’s next steps in the immediate postdischarge period - Links and QR codes to online resources about recovery and CR (<https://www.monash.edu/medicine/rural-health/research/projects/equal-hearts/equal-hearts-resources>) are also provided on the action plan. |
| 1. **WHAT: Procedures**   Describe each of the procedures, activities and/or processes used in the intervention, including any enabling or support activities | The Patient Discharge Action Plan is intended to be filled out by the patient and CR clinician prior to leaving the hospital. This intervention is delivered in addition to usual care, meaning that participants in the intervention group will receive all education that is provided as part of usual care, plus the Patient Discharge Action Plan. |
| 1. **WHO PROVIDED**   For each category of intervention provider (e.g., psychologist, nursing assistant), describe their expertise, background and any specific training given | The intervention will be delivered by CR clinicians as part of their normal role on the cardiac wards and who have attended training specific to the intervention prior to commencement. |
| 1. **HOW**   Describe the modes of delivery (e.g., face to face or by some other mechanism, such as the internet or telephone) of the intervention and whether it will be provided individually or in a group | The intervention will be delivered individually and face-to-face. |
| 1. **WHERE**   Describe the type of location where the intervention occurred, including any necessary infrastructure or relevant features | Participants were recruited from public inpatient rooms in a cardiac hospital service in Melbourne, Australia. |
| 1. **WHEN and HOW MUCH**   Describe the number of times the intervention was delivered and over what period of time including the number of sessions, their schedule, duration, intensity or dose | The Discharge Patient Action Plan will be delivered to patients when they receive their normal discharge education. The plan will be delivered within 48 hours prior to discharge. The plan is designed to be completed by the CR clinicians in collaboration with the patient, who then takes it home. It will take approximately 15-30 min in addition to usual discharge education. |
| 1. **TAILORING**   If the intervention was planned to be personalized, titrated or adapted, then describe what, why, when and how | The action plan is personalized for each patient with a section that addresses the patient’s individual concerns (e.g. about driving, exercise, work, etc.) and a section addressing any questions or concerns. |
| 1. **MODIFICATIONS**   If the intervention was modified during the course of the study, describe the changes (what, why, when and how) | Not applicable at this stage. |
| 1. **HOW WELL: Planned**   If intervention adherence or fidelity was assessed, describe how and by whom, and if any strategies were used to maintain or improve fidelity, describe them | An intervention fidelity plan has been developed. |
| 1. **HOW WELL: Actual**   If intervention adherence or fidelity was assessed describe the extent to which the intervention was delivered as planned | Not applicable at this stage. |
